# Intraosseous Mucoepidermoid Carcinoma: Report of Two Cases

**Published:** 2014-06

**Authors:** S. Atarbashi Moghadam, F. Atarbashi Moghadam

**Affiliations:** a Dept of Oral & Maxillofacial Pathology, Shahid Beheshti University of Medical Sciences, Tehran, Iran.; b Dept. of Periodontics, Shahid Sadoughi University of Medical Sciences, Yazd, Iran.

**Keywords:** Intraosseous, Central, Carcinoma mucoepidermoid, Odontogenic cyst

## Abstract

Intraosseous mucoepidermoid carcinoma is a rare tumor which affects women more than men and is more common in the mandible. The radiological examination reveals a well-defined unilocular or multilocular radiolucent lesion. This tumor may resemble a glandular odontogenic cyst, particularly in incisional biopsies. The accurate diagnosis of these lesions is imperative because the subsequent treatment of each lesion would be different. The purpose of this study is to report two cases of intraosseous mucoepidermoid carcinoma and explicate the differentiating criteria of this lesion from the glandular odontogenic cyst.

## Introduction

Intraosseous mucoepidermoid carcinomas (MEC) of the jaws are rare, establishing 2-3% of all testified mucoepidermoid carcinomas in the literature [1-2]. This tumor may originate from ectopic salivary gland tissue or may have been instigated by transformation of mucous cells found in odontogenic cysts and maxillary sinus or submucosal salivary glands having intraosseous extension [1, 3].

Intraosseous MEC affects females more than males and apparently implicates the mandible more than the maxilla [4]. It may be challenging to determine whether a maxillary tumor is raised initially within the maxillary bone or it only represents the central extension of a neoplasm which has originated within the sinus mucosa [5].

It has been reported in all age; it is more prevalent in 4th and 5th decades of life [4]. Various radiological patterns have been reported which seems to be non-diagnostic or barely helping in definitive diagnosis and therefore, biopsy appears to be crucial for the definite diagnosis [5]. Generally, the prognosis of intraosseous MEC is good [6]. Wide local excision is the foremost modality of treatment and the recurrence rate of 40% was conveyed after conservative surgery as the treatment. Radiotherapy, as the consolidating therapy, is recommended for high grade tumors [4].

Glandular odontogenic cyst (GOC), a rare lesion, constitutes 0.2% of all odontogenic cysts [7]. This tumor, most likely, occurs in the mandible with a tendency to the anterior region [8]. Asymptomatic swelling or expansion is the most prevalent clinical verdict. Radiographically, the lesions are often exhibited as well-defined, unilocular or multilocular radiolucency. Enucleation, curettage and the local block excision have been described as the different treatment modalities for GOC. The prognosis is stated to be good; however, this lesion has a high rate of recurrence which subsequently necessitates a long-term follow up after the relevant treatment [7-8]. Microscopically, GOC might be confused with intraosseous MEC [9] and many reports are available regarding the microscopic similarities of these two lesions and therefore the possible consequent misdiagnosis [5-9]. The correct diagnosis is imperative since these two lesions entail different treatment plans [10]. Immunohistochemistry and gene abnormality evaluation tests might be helpful for differential diagnosis [10-11].

The purpose of this report was to depict two cases of intraosseous mucoepidermoid carcinoma and elucidate the possible ways to differentiate this lesion from a glandular odontogenic cyst. 

## Case 1

**Figure 1:**
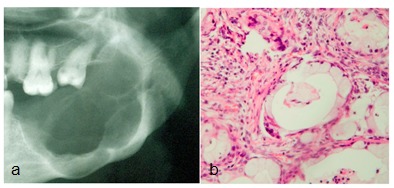
**a:** Radiographic image revealed a large, well-defined, multilocular radiolucent lesion with scalloped borders extending from the first molar area into the ascending ramus with cortex perforation.  **b:** Histopathological exam showed islands of epidermoid and mucous cells, with cystic areas in a fibrous stroma (X400).

A 44-year-old woman was referred to Jahad Clinic- Ahvaz, Iran, exhibiting a painless swelling in the left posterior region of the mandible endured for 2 years. The entire molar teeth were extracted in this region. The radiological examination illustrated a large, well-defined, multilocular radiolucent lesion with scalloped borders extending from the first molar area into the ascending ramus (Figure 1a); which perforated the cortex.

No palpable lymph node was noticed in the neck on extraoral examination. A provisional diagnosis of odontogenic tumors was verified and subsequently an incisional biopsy was performed. The microscopic examination revealed islands of epidermoid, mucous and intermediate cells with cystic areas in a fibrous stroma (Figure 1b). The final diagnosis was established as intraosseous low-grade mucoepidermoid carcinoma. The patient was treated with hemi-mandibulectomy.

## Case 2

**Figure 2:**
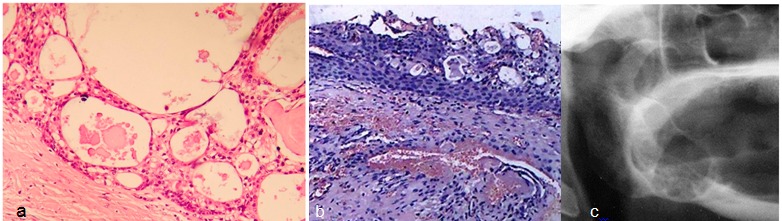
**a:** Radiographic examination revealed a large, well-defined, multilocular radiolucent lesion extending from the posterior body of mandible into the ascending ramus. **b:** The microscopic sections showed a cystic lesion lined by stratified squamous epithelium with small microcysts (X100)  **c:** Histopathology demonstrated nests and islands of epidermoid and mucous cells in a fibrous stroma with many cystic spaces (X200).

A 56-year-old edentulous woman was referred to Jahad clinic, Ahvaz, Iran,  complaining of a painless expansion of right posterior mandible endured for 11 months. Radiological assessment illustrated a large, well-defined multilocular radiolucent lesion, extending from the posterior body of the mandible into the ascending ramus (Figure 2a). On extraoral examination, no palpable lymph node was detected in the neck region. Therefore, the incisional biopsy was performed regarding the initial diagnosis of ameloblastoma. The microscopic sections displayed a cystic lesion lined by stratified squamous epithelium, exhibiting small microcysts and numerous clusters of mucous cells. Concerning these features, the diagnosis of glandular odontogenic cyst (GOC) was rendered (Figure 2b). The lesion was excised completely and the excisional biopsy revealed nests and islands of epidermoid and mucous cells in a fibrous stroma with many cystic spaces (Figure 2c). The ultimate diagnosis was confirmed as intraosseous low-grade mucoepidermoid carcinoma and the patient was referred for additional treatments such as hemi-mandibulectomy with condylar preservation.

## Discussion

The criteria that acknowledges the diagnosis of intraosseous MEC is entailed as: (a) intact cortical plates while the cortical perforation does not exclude the diagnosis, (b) radiological evidence of bone destruction, (c) exclusion of an alternative primary tumor, which its metastasis could histologically resemble the central MEC, (d) exclusion of an odontogenic tumor and (e) histopathologic confirmation [2, 5]. In the current study, the patients possessed all the necessary criteria. The clinical presentation consists of pain, swelling and altered nerve sensation in a long-standing lesion; however, the common symptom in the current cases was swelling; which was in agreement with the findings of the study presented by He et al. [1].

The radiological features are diverse and presumably non-diagnostic, usually presented as a unilocular or multilocular radiolucency. Even though it is frequently scalloped; the margins of the lesion are often well defined [5]. To the best of our knowledge, only a few cases of mixed radiolucent-radiopaque lesions have been reported in the literature [12-16].

Chan et al. [17] stated that the common radiological features of these tumors would be the presence of a well-defined sclerotic boundary, internal amorphous sclerotic bone and many small loculations. Moreover, bordering septa in many of these loculations is absent and the outer cortical plate is expanded and perforated with extending into the surrounding soft tissue. Tooth displacement and root resorption is also present. All reported cases of their study exhibited some common diagnostic imaging aspects with other multilocular-appearing lesions of the jaws. Though, the presence of amorphous sclerotic bone and malignant features can be advantageous in the differential diagnosis [17].

The presence of calcifications was reported in the clear-cell variant and the conventional MEC, occurring in enduring neoplasms. The dystrophic calcification of the amorphous-eosinophilic material secreted by intermediate basal cells may perhaps produce these features [12-15]. Eversole et al. [18] found that 50% of mandibular central MEC were associated with dental cysts or impacted teeth; whereas in the study of Brookstone and Huvos [19] and in the research of He et al. [1], no significant relationship was found between central MEC and odontogenic cysts or impacted teeth. Likewise, no relationship was detected between impacted tooth and central MEC in our study. Microscopic examination of MEC revealed a neoplasm composed of nests and islands of epidermoid, mucous, and intermediate cells embracing cystic spaces with various sizes in a fibrous connective tissue [4]. A considerable number of central MEC have been reported to be mainly low-grade cystic lesions [5]. The presenting two cases were also low grade.

Histopathologically, GOC may be confused with intraosseous MEC [7-9]. This cyst is lined by stratified squamous epithelium with variable thickness and surface cuboidal or columnar ciliated cells. Small microcysts and clusters of mucous cells are also depicted [10]. Islands, resembling intraosseous MEC, were noted in the GOC wall; which may possibly cause diagnostic drawback [9]. Therefore, molecular assays, explicitly targeting the MEC-like islands in the GOC fibrous wall, may figure out whether these islands signify true malignant transformation or not [6, 8, 10]. In the current study, the second case was primarily diagnosed as GOC. Inevitably, the incorrect diagnosis proceeds to patient complications and overdue treatment. This is imperative since different treatments are demanded for patients with a GOC compared to low- grade MEC. Supplementary implements and methods such as immunohistochemistry and gene abnormality assessments might be supportive in yielding a conclusive differential diagnosis [10-11]. One of these markers is Maspin which is expressed in MEC and would be expedient in the differential diagnosis of MEC from GOC, particularly in ambiguous cases and in small incisional biopsy samples [10].

Pires et al. [11] stated that the origin of central MEC is still controversial. GOC is a newly defined entity whose association with low-grade central MEC has been described in the literature. Moreover, the study of Pires et al. was aimed to evaluate the cytokeratin (CK) profile of central MEC and GOC, matching the outcomes with the expression of CK in MEC of salivary glands and odontogenic cysts and tumors. They concluded that all central MECs expressed CKs 5, 7, 8, 14, and 18 and all GOCs expressed CKs 5, 7, 8, 13, 14, and 19 [11].

They compared CK expression in GOC and central MEC and noticed dissimilarities in CKs 18 (30% versus 100%) and 19 (100% versus 50%). Central MEC and GOC are perhaps distinct lesions with CK profiles comparable to lesions that have glandular and odontogenic origins, respectively. Moreover, expression of CKs 18 and 19 could be beneficial in their subsequent differential diagnosis [11].

TORC1/MAML2 and MECT1:MAML2 gene fusion in intraosseous MEC was reported by studies of Khan et al. [6] and Fowler et al. [7] and it has been confirmed that they can be employed as a diagnostic marker [6-7].

The foremost treatment for intraosseous MEC is wide local resection, enblock resection or hemi-mandibulectomy [1, 20]. Selective or therapeutic neck dissection has been introduced in the instances of cervical lymphatic metastasis [1].

Radiotherapy seems to be a useful supplementary aid in cases represented with close surgical margins and high grade tumors [20]. Microscopic grading appears to have a strong influence on survival rate; so that a low-grade tumor without perineural invasion and with tumor-free margins designates a better prognosis [21].

In He et al.’s study [1], all patients presented low- grade tumors without any evidence of nodal metastasis. The current case similarly did not demonstrate any cervical  lymphadenopathy. Brookstone and Huvos [19] have proposed a staging class for intraosseous MEC. Lesions with intact cortical plates with no evidence of bone expansion are categorized as stage I; neoplasms with intact plates, but intrabony expansion are branded as stage II and finally, lesions associated with cortical perforation or nodal disease are classified as staged III. According to these categories, in the current study, the case 1 can be sorted in stage III and case2 can be fitted in stage II.

The recurrence rate of this entity varies from 13-50% in different studies [20]. Metastases are reported in 9% of central MEC primarily to the regional lymph nodes and infrequently to the ipsilateral clavicle, lung and brain, hence, long term follow up is recommended [4].

## Conclusion

Intraosseous MEC may resemble GOC; therefore, pathologists and surgeons should deliberate the diagnosis of central MEC in their mind once the pathology report signifies GOC. This is particularly imperative when the lesion is located in the posterior of the mandible. Although many case reports outlines the radiological features of this entity, the diagnosis should be established on clinical and pathologic characteristics and local or radical resective surgery would probably be the first option for patient treatment. Immunohistochemistry might be useful in the diagnosis of complicated cases or small specimens. Concerning the diagnosis of GOC, obtaining serial sections of the lesion is highly recommended. 
